# Histology to 3D in vivo MR registration for volumetric evaluation of MRgFUS treatment assessment biomarkers

**DOI:** 10.1038/s41598-021-97309-0

**Published:** 2021-09-23

**Authors:** Blake E. Zimmerman, Sara L. Johnson, Henrik A. Odéen, Jill E. Shea, Rachel E. Factor, Sarang C. Joshi, Allison H. Payne

**Affiliations:** 1 Department of Biomedical Engineering, University of Utah, Salt Lake City, UT USA; 2 Scientific Computing and Imaging Institute, University of Utah, Salt Lake City, UT USA; 3 Utah Center for Advanced Imaging Research, University of Utah, Salt Lake City, UT USA; 4 Department of Surgery, University of Utah, Salt Lake City, UT USA; 5 Department of Pathology, University of Utah, Salt Lake City, UT USA

**Keywords:** Biomedical engineering, Preclinical research, Magnetic resonance imaging, Targeted therapies, Applied mathematics

## Abstract

Advances in imaging and early cancer detection have increased interest in magnetic resonance (MR) guided focused ultrasound (MRgFUS) technologies for cancer treatment. MRgFUS ablation treatments could reduce surgical risks, preserve organ tissue and function, and improve patient quality of life. However, surgical resection and histological analysis remain the gold standard to assess cancer treatment response. For non-invasive ablation therapies such as MRgFUS, the treatment response must be determined through MR imaging biomarkers. However, current MR biomarkers are inconclusive and have not been rigorously evaluated against histology via accurate registration. Existing registration methods rely on anatomical features to directly register in vivo MR and histology. For MRgFUS applications in anatomies such as liver, kidney, or breast, anatomical features that are not caused by the treatment are often insufficient to drive direct registration. We present a novel MR to histology registration workflow that utilizes intermediate imaging and does not rely on anatomical MR features being visible in histology. The presented workflow yields an overall registration accuracy of 1.00 ± 0.13 mm. The developed registration pipeline is used to evaluate a common MRgFUS treatment assessment biomarker against histology. Evaluating MR biomarkers against histology using this registration pipeline will facilitate validating novel MRgFUS biomarkers to improve treatment assessment without surgical intervention. While the presented registration technique has been evaluated in a MRgFUS ablation treatment model, this technique could be potentially applied in any tissue to evaluate a variety of therapeutic options.

## Introduction

Improved early cancer detection has driven the development of more conservative, less invasive cancer treatment alternatives to surgical intervention. These minimally and non-invasive treatments have the potential to reduce or eliminate surgical risks, preserve organ tissue and function, and improve patient quality of life. Magnetic resonance (MR) guided focused ultrasound (MRgFUS) ablation is a rapidly growing technology for non-invasive disease treatment with applications including benign^1^ and malignant tumors^2^, neurological disorders^3^, control localized drug delivery^4^, opening the blood brain barrier^5,6^, and modulation of nerve functionality^7–9^. While MRgFUS is FDA approved to treat essential and Parkinsonian tremor, bone metastases, prostate disease and uterine fibroids, it is not yet approved to treat cancer. Because the tumor or other treated tissue is not resected during MRgFUS therapy, treatment response must be determined non-invasively through imaging biomarkers. Therefore, gold-standard determination of MRgFUS treatment response initially requires tissue resection and histological analysis^10,11^. The most robust way to validate MR biomarkers is through spatially accurate and precise comparison between in vivo MR biomarkers and histology.

Directly comparing MR images of in vivo tissue (in vivo MR) to histology is challenging because processing excised tissue for histological analysis can substantially distort the tissue as well as destroy the spatial relationship between in vivo MR and histology images^12^. Because of this difficulty, previous studies have not restored this spatial relationship and evaluated MRgFUS MR biomarkers via qualitative or indirect metrics, such as non-viable tumor fractions that can be derived relative to the MR and histology spaces independently^13^. Qualitative analysis and indirect metric evaluation have been used to evaluate several MR biomarkers, including the commonly used non-perfused volume (NPV) measured on contrast-enhanced (CE) T1-weighted (T1w) MR imaging and other biomarkers such as T2w imaging, thermal dose, and diffusion imaging^4–17^. Although the current spatially non-specific qualitative analysis and indirect metrics demonstrate a strong correlation between some MR biomarkers and histology, conclusive spatial accuracy cannot be determined with these methods. For example, conflicting studies show that the NPV biomarker assessed immediately ($\sim$ 1 h) after treatment both over- and under estimates histology lesion size^11,18^; however, NPV has increased correlation with histology lesion size several days post treatment^11,19^. To enable quantitative spatial comparison of 3D MR and volumetric histological data, the spatial relationship between in vivo MR and histology must be restored to evaluate the effectiveness and establish the predictive accuracy of in vivo all MR imaging biomarkers.

Several studies have developed MR to histology registration methods to restore the spatial relationship between in vivo diagnostic or treatment evaluation MR images and 2D histology imaging^10,20–26^. With accurate registration, in vivo MR biomarkers can be directly compared to histology using a variety of spatial metrics. The required degree of registration accuracy depends on the resolution of the biomarker being evaluated. Current in vivo MR biomarkers are typically acquired with an in-plane resolution of 1–2 mm, requiring less than one millimeter accuracy for accurate evaluation^11,27^. A registration technique with this level of precision would allow for the rigorous evaluation of the novel imaging biomarkers being developed by the research community. Some existing registration methods rely on features that correlate directly between in vivo MR images and histology that are independent of the diagnostic or treatment features being evaluated^25,28^. These independent features can be used to facilitate registration without influencing the final comparison between MR treatment features and the corresponding histology response. For example, registration methods for prostate applications excise the entire organ and use whole-mount histology to allow a correlation between the organ boundary on both in vivo MR and histology^20,21,29–31^. Additional registration methods rely on correlating anatomical landmarks to support the registration process^10^. However, for many MRgFUS ablation applications including liver, breast, or kidney, anatomical features independent from the ablation treatment features are often not available for input to registration algorithms. This lack of sufficient correlating features between in vivo MR and histology independent of the treatment features prohibits the use of previously developed registration techniques. While potential treatment dependent features are often visible on both in vivo MR and histology, the use of these features to drive registration can bias the final result of biomarker evaluation. Therefore, there is a critical need for a new registration method that uses intermediate imaging steps in lieu of feature correlation to account for the deformations incurred at every step of the histology tissue preparation process, allowing the accurate registration of in vivo MR and histology to evaluate MRgFUS treatment imaging biomarkers. This rigorous, spatially accurate registration technique will enable the development of the rigorously validated imaging biomarkers required for the non-invasive treatment of cancer with MRgFUS.

In this paper, we present a novel, multi-step MR to histology registration workflow that corrects all deformations without assuming any direct feature correlation between in vivo MR and histology, but rather between three intermediate stages. Deformations from every tissue processing step are estimated and composed to generate a full 3D map between any histology image and in vivo MR biomarkers. The histology space is densely sampled and histology annotations are mapped to in vivo MR to form a volumetric histological annotation of tissue necrosis in the in vivo MR space. This novel registration workflow facilitates a rigorous quantitative evaluation of MR imaging biomarkers that was not possible before, allowing use of clinically relevant spatial metrics such as precision, recall, DICE coefficient, and Hausdorff distance^32^. We demonstrate the capabilities of this registration pipeline by evaluating the NPV biomarker following MRgFUS ablation of a VX2 rabbit tumor model at two time points: (1) acute NPV ($\sim$ 1 h after ablation) and (2) post-NPV (3–5 days after treatment). We compare these spatially quantified results to the findings of qualitative and indirect findings in the literature. The presented registration method will facilitate accurately validating a wide range of in vivo MR biomarkers against the gold standard of histology for non-invasive MRgFUS therapies. Validating in vivo MR biomarkers will increase their clinical viability for MRgFUS treatment assessment and facilitate minimally invasive treatments becoming feasible alternatives to surgical intervention for cancer treatments.

**Figure 1:**
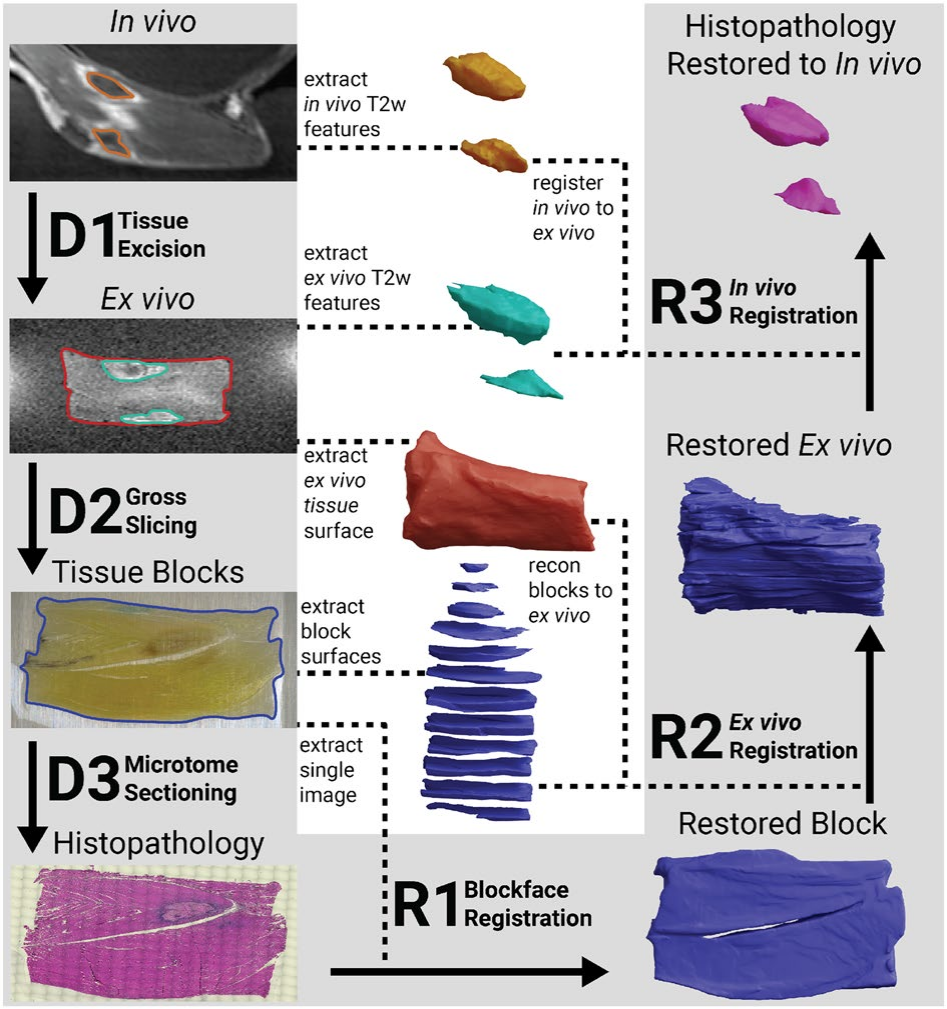
Flow chart of the end-to-end workflow required to register in vivo MR to histology. The steps for destructive histopathology pipeline are indicated by D1, D2, and D3, and the steps for restoring registration pipeline are indicated by R1, R2, and R3. Dashed lines indicate information extracted from digital imaging during the destructive histopathology pipeline for use in the restoring steps.

## Overview of approach

**Figure 2:**
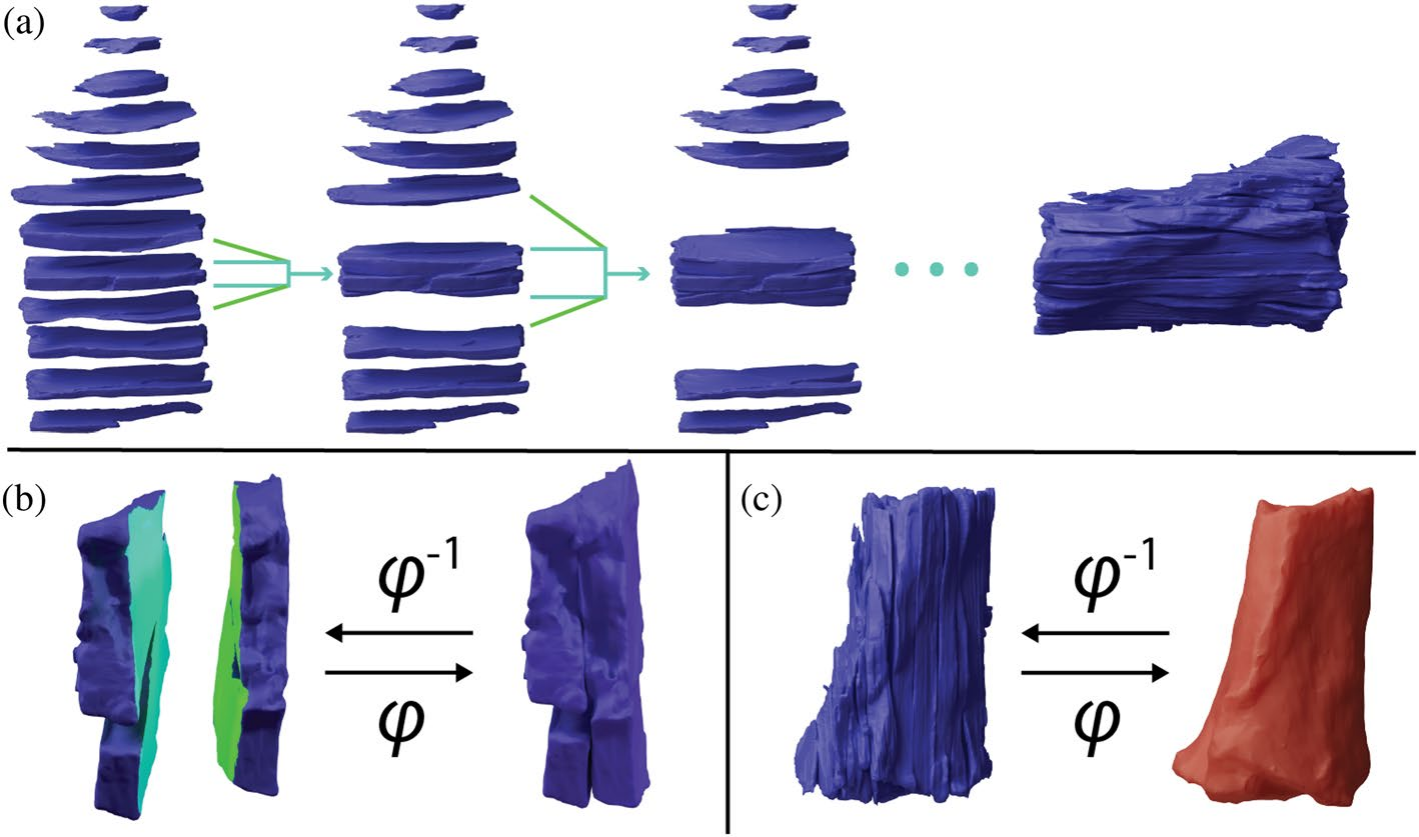
Depiction of the block reconstruction process. The sequential block reconstruction is shown in **(a)**. Two blocks are registered together with a 3D, nonlinear transform using corresponding faces as in **(b)**, where the light blue face is the target face and does not move, and the light green face is the moving face. This registration results in a transformation $\varphi$ for the moving block. The reconstructed block is registered to the red ex vivo surface in **(c)** to generate a transformation $\varphi$ between the reconstructed blocks and ex vivo MR imaging.

The presented registration approach utilizes intermediate imaging to comprehensively correct for deformations introduced throughout the tissue processing workflow. Intermediate imaging provides digital representations of the tissue both before and after specific deformations have been introduced during processing, eliminating the need for correlating features between in vivo MR and histology to drive the registration, as has been done in previous methods. A depiction of the general workflow is shown in Fig. 1. The pipeline developed and described in this work combines previously developed registration strategies and contributes novel registration methods^10^. The tissue processing and registration pipelines were evaluated on four subjects of a large animal tumor model described in “Subject model”.

The (D)estructive histopathology pipeline seen in Fig. 1 consists of three main steps that introduce deformation into the tissue: tissue excision (D1), gross slicing (D2), and microtome sectioning (D3). Surgical excision and formalin fixation of the treated tissue introduces orientation uncertainty and non-linear deformation into the tissue sample. The excised tissue is typically too large to be processed as a whole, so the sample is grossly sliced into smaller tissue blocks, usually $\sim$3–5 mm. The gross slicing step introduces non-linear deformations to each tissue block and destroys the spatial relationship between each block and the original volume. In typical histology processing, after paraffin wax embedding, each block face is trimmed using a microtome until a full section of tissue is exposed (’facing the block’), and then sections are collected for histology and the remaining tissue is set aside^10^. However, the presented method requires microtome sectioning through the entire tissue block, collecting sections for histology throughout the block. Microtome sectioning and subsequent staining and mounting cause non-linear deformations from tissue shearing, tearing, and stretching. The end product of the destructive histopathology pipeline is a series of stained 2D microscopic images that must be re-aligned with in vivo MR.

The (R)estoring registration pipeline independently estimates the deformation between each of three main destructive histopathology steps via blockface registration (R1), ex vivo registration (R2), and in vivo registration (R3). Blockface registration (R1) estimates the 2D deformations from microtome sectioning, staining, and mounting. After blockface registration, each 2D histology image must be correlated with the 3D tissue volume before gross slicing. In this step, most other registration methods depend on 2D slice correlation between MR (in vivo or ex vivo) and histology to restore the relationship between the original tissue volume and histology^10^. However, our novel ex vivo registration (R2) step uses 3D models from ex vivo MR and blockface imaging to embed each histology section into the original 3D tissue volume without assuming slice-to-slice correspondence or correlation between MR and histology features. A general overview of this reconstruction step is shown in Fig. 2. We emulate the 2D slice correspondence assumption in our ex vivo registration step and compare the error of the presented method against this assumption to show the benefit of the 3D model reconstruction. Finally, in vivo registration (R3) estimates deformations from tissue excision by registering 3D models of corresponding features from ex vivo and in vivo MR images. The deformations between any two steps is represented by a diffeomorphism, and these deformations can be composed to generate a single diffeomorphism between multiple stages of imaging. A single diffeomorphism from histology to in vivo MR inserts each histology section into the in vivo MR space and enables the generation of fully registered, volumetric histological necrosis annotations in the in vivo MR space.

The necessary accuracy of registration is dependent on the native resolution of the evaluation space, which is in vivo MR imaging. Ideally, the registration accuracy should be at or below the resolution of the acquired MR biomarkers being evaluated. Common clinical biomarkers are acquired with an in-plane resolution of 1–2 mm, making 1 mm of total error an ideal constraint for the total registration error^11,27^. The accuracy of the presented registration pipeline was evaluated at every stage using the Euclidean distance between corresponding landmarks following registration, known as the target registration error (TRE). These landmarks are only between intermediate stages, not directly between in vivo MR and histology, and are not used to drive registration. For example, features to evaluate R3 between in vivo T2w MR and ex vivo T2w MR can be identified because they are the same imaging modality. Additionally, landmarks, such as the the edge of the tissue, can be selected between histology sections and blockface images to evaluate R1. However, the edge of the tissue is not identifiable on in vivo MR (because the tissue was trimmed) and the appearance of the T2w MR features in histology may not be known. Unlike other applications, selecting landmarks that correlate between in vivo MR and histology is not possible in this application, so we evaluate the individual stages of registration. The total accuracy of the pipeline is estimated by summing the errors from each stage to obtain the cumulative error from registration. To determine the potential impact of the 2D slice projection on the registration accuracy, the landmarks from ex vivo registration (R2) are deformed using the proposed registration method and the 2D slice assumption method independently and compared. The acute NPV and post-NPV biomarkers are spatially evaluated against the volumetric histological annotation of necrosis resulting from registration using spatially specific metrics, including the precision, recall, DICE coefficient, and Hausdorff distance.

**Figure 3:**
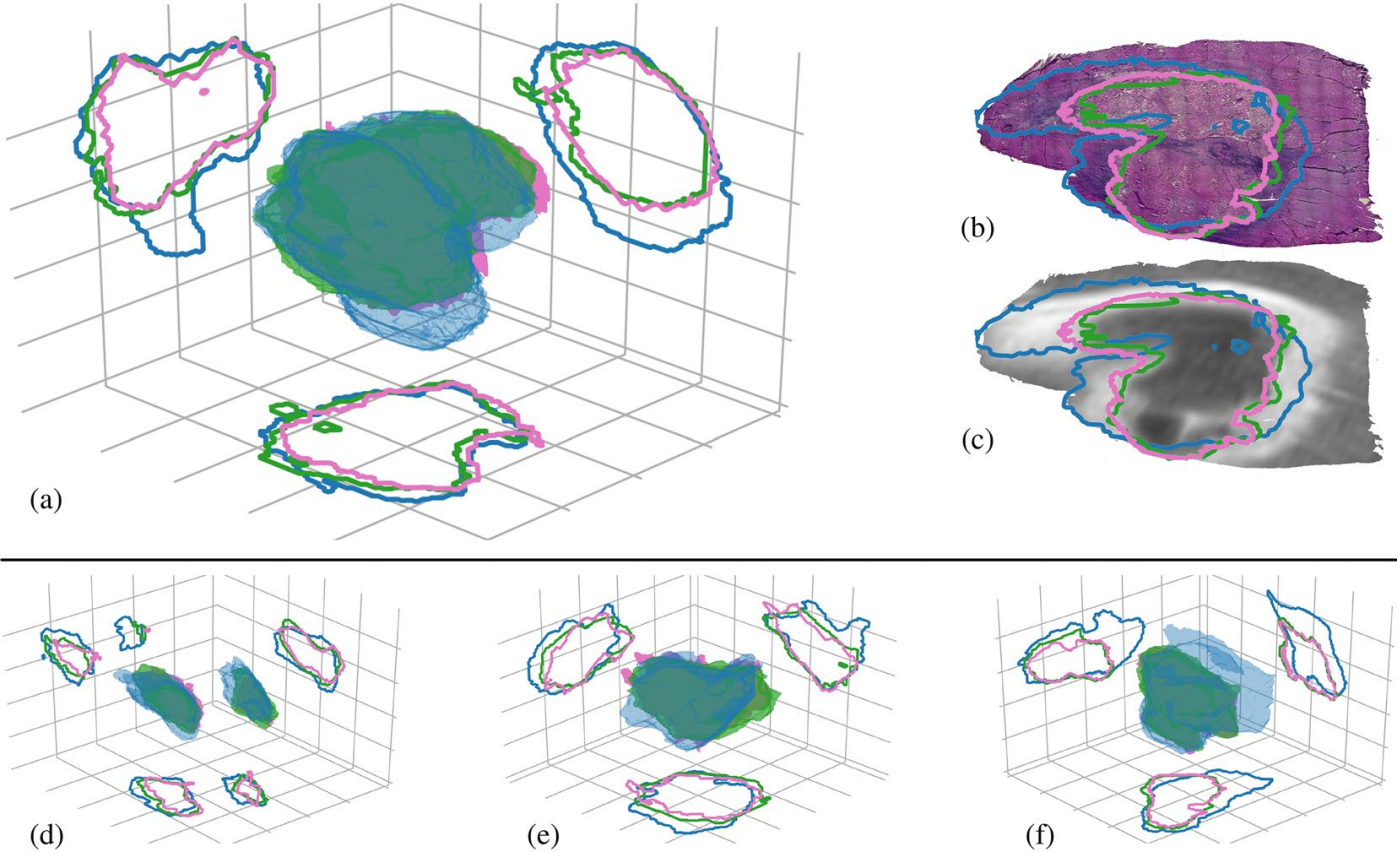
MR biomarker to histology registration with accurate spatial comparisons of the acute NPV (blue), post-NPV (green), and histology necrosis (pink) labels. **(a,d–f)** show volumetric overlays of each of the labels in the *in vivo* MR space for all four subjects , **(a)** 1, **(d)** 2, **(e)** 3, and **(f)** 4, respectively. The contours on the axis walls are projections for each respective volume from the slice with the largest acute NPV area in each dimension. The space between each grid line is 5 mm. **(b)** Label contours from the subject shown in **(a)** sampled into the native histology space. **(c)** Follow-up contrast-enhanced MR image resampled onto the corresponding histology slice in **(b)** with the each of the labels overlaid, demonstrating that the estimated transformations are invertible. The time duration between the acute- and post-NPV is detailed in Table 2.

## Results

**Figure 4:**
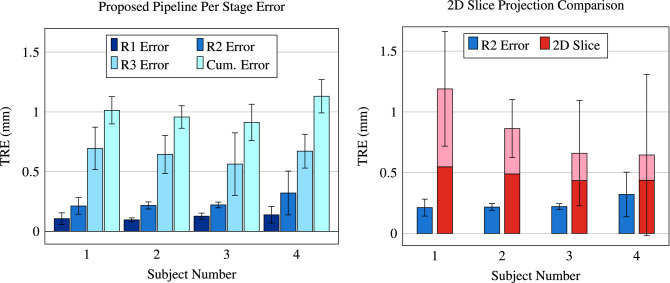
Target registration error (TRE) computed on a subject-specific basis. (Left) Subject-specific TREs for the three stages of the registration pipeline: blockface (R1), ex vivo (R2), and in vivo (R3) registration. The cumulative error is the summation of the mean of three stages for each subject. (Right) Subject-specific TREs from ex vivo registration (R2) for the presented method versus the 2D slice correlation method. The dark region of the 2D slice bar shows the portion of the total error resulting only from the Z dimension.

The goal of the registration pipeline was to provide a 3D volumetric label of histological necrosis registered to the in vivo MR imaging space. Figure 3 includes a volumetric comparison between the acute NPV (blue) and post-NPV (green) MR biomarkers and the histology necrosis (pink) for each of the four subjects analyzed. The histology volume represents the label of tissue necrosis against which the acute and post NPV MR biomarkers were independently compared to determine their accuracy. The spatial metrics calculated from these volumes are presented in Table 1. The TRE accuracy for each stage and specific stages under different assumptions is displayed in Fig. 4.

**Table 1:** The total volume and different spatial evaluation metrics for each label, the acute NPV and post-NPV, compared against the histology volume for each subject analyzed.

#	Acute NPV vol. (mm$^3$)	Post NPV vol. (mm$^3$)	Histology vol. (mm$^3$)	Acute NPV precision	Post NPV precision	Acute NPV recall	Post NPV recall	Acute NPV DICE	Post NPV DICE	Acute NPV Hausdorff (mm)	Post NPV Hausdorff (mm)
1	1317.35	915.74	675.59	0.39	0.62	0.76	0.85	0.52	0.72	7.61	3.17
2	2039.13	1688.73	1281.84	0.49	0.69	0.78	0.91	0.60	0.78	6.09	3.31
3	3859.13	2627.20	2391.17	0.49	0.86	0.80	0.94	0.61	0.90	7.51	3.05
4	5542.83	3969.91	3477.31	0.58	0.81	0.92	0.92	0.71	0.86	12.14	3.07

### Registration pipeline accuracy

The accuracy of each stage of the registration pipeline was independently evaluated using landmarks; the R1 landmarks were different from the R2 landmarks, which were both different from the R3 landmarks. TREs for the different stages of the registration pipeline are plotted in Fig. 4 for the four subjects analyzed. The TRE for blockface registration (R1), ex vivo registration (R2), and in vivo registration (R3) ranged from 0.096 to 0.139 mm (mean ± s.d. = 0.117 ± 0.045 mm), 0.21 to 0.32 mm (0.24 ± 0.10 mm), and 0.56 to 0.69 mm (0.64 ± 0.19 mm), respectively. The sum of the error for the different stages ranged from 0.91 to 1.13 mm (1.00 ± 0.13 mm) for the four subjects analyzed.

### Comparison with 2D slice correlation

The ex vivo registration (R2) was run while restricting the Z dimension (perpendicular to the histology images) motion to only translation, enforcing the assumption that the blockface and 2D histology slices correlate with an ex vivo MR slice. The TRE for the presented ex vivo registration method and the 2D slice correlation method can be seen in Fig. 4. The presented method ranged from 0.21 to 0.32 mm (0.24 ± 0.10 mm) and significantly (p $=$ 0.025, N $=$ 20) outperformed the 2D slice correlation method, which ranged from 0.65 to 1.19 mm (0.84 ± 0.22 mm). The 3D error from the presented method even significantly (p $=$ 0.015, N $=$ 20) outperformed the Z dimension error of the 2D slice correlation method alone, ranging from 0.44 to 0.54 mm (0.48 ± 0.05 mm).

### NPV biomarker evaluation

The total volume of each label and the spatial metrics comparing the acute NPV and the post NPV MR biomarkers against the histology necrosis label are shown in Table 1. The post-NPV precision significantly outperformed the acute NPV precision (0.0079, N $=$ 4) when compared to the histology necrosis labels, ranging from 0.62 to 0.86 (0.74 ± 0.09) and 0.39 to 0.58 (0.49 ± 0.07), respectively. The post-NPV recall ranged from 0.76 to 0.94 (0.91 ± 0.03) but was not significantly different (p $=$ 0.079, N $=$ 4) from the acute NPV that ranged from 0.76 to 0.92 (0.82 ± 0.06). The DICE similarity coefficient was significantly better (p $=$ 0.011, N $=$ 4) between post-NPV and histology, which ranged from 0.72 to 0.9 (0.82 ± 0.07), than between the acute NPV and histology, which ranged from 0.52 to 0.71 (0.61 ± 0.07). Finally, the Hausdorff distance for post-NPV ranged from 3.05 to 3.31 mm (3.15 ± 0.10 mm) and was significantly smaller (p $=$ 0.007, N $=$ 4) than the Hausdorff distance for the acute NPV, ranging from 6.09 to 12.14 mm (8.34 ± 2.28 mm).

## Discussion

The rigorous spatial evaluation of the acute and post-NPV MR biomarkers against the gold-standard histology label is only possible because of the presented MR to histology registration method. This evaluation revealed that NPV biomarker acquired 3–5 days after treatment was a more accurate predictor of the MRgFUS induced tissue necrosis than the acute NPV. Although this result is consistent with prior studies that have used qualitative or indirect metrics to compare NPV and histology^11,18^, the presented results quantitatively demonstrate the significant difference between the two metrics. The acute NPV over predicted the histology label for every subject, as can be seen in Fig. 3, reinforced by the low precision score, high recall score, and larger volume. Looking at the contour comparisons in Fig. 3, the acute NPVs for subjects 2, 3, and 4 have distinct features that are not present in the histology label. These large, mislabeled regions are reflected in the large Hausdorff distances between the acute NPV and histology, with the most obvious mislabeled structure in subject 4 with a Hausdorff distance of 12.14 mm. These regions and general overestimation of the acute NPV could be a result of transient effects previously described in the literature, such as vascular occlusion^11^. These results demonstrate the acute NPV biomarker measured on CE-T1w MR imaging is not an accurate predictor of histological tissue viability following MRgFUS ablation for the presented model.

The post-NPV MR biomarker significantly outperformed the acute NPV in every spatial metric except for recall. This result is expected because the overestimation yields a high recall score, which explains the lack of a significant difference between the acute NPV and the post-NPV recall. However, the mean precision of the post-NPV biomarker was significantly higher, indicating that the post-NPV biomarker had fewer false positives compared to the histology label than the acute NPV. The qualitative alignment of the post-NPV to histology volume in Fig. 3 is quantitatively reflected in the significantly lower and more consistent reported Hausdorff distances relative to the acute NPV. Therefore, with these metrics, it can be deduced that the acute NPV overestimates the necrotic volume and while the post NPV improves the prediction of the necrotic volume when compared to the acute NPV, it still overestimates the true necrotic region. It is important to note that these spatial metric results cannot be used to evaluate the accuracy of the registration because the post-NPV MR biomarker may not perfectly match the histology volume even with perfect registration. These correlations between in vivo MR and histology are what we aim to understand and therefore cannot be used to drive or evaluate the registration without biasing the final result.

The average TRE of 1.00 ± 0.13 mm resulting from the presented registration workflow is lower than the TRE reported by state-of-the-art MR to histology registration methods used in similar applications^26,30^. The TRE of the presented registration workflow is on the order or the resolution of several clinical MR biomarkers, making the workflow suitable for evaluating these biomarkers^11,27^. Additionally, the reported registration accuracy is achieved without relying on features that correlate directly between in vivo MR images and histology that are independent of the diagnostic or treatment features being evaluated. R3 registration between in vivo MR and ex vivo MR in the presented method may include treatment features. Correlating features between in vivo and ex vivo MR is possible because the image contrast is consistent, and the deformation is minimal between the two stages. However, the direct relationship between in vivo and the H&E stained tissue sections is unknown, so features between in vivo MR and histology may or may not directly correlate. Therefore, selecting features directly between in vivo MR and histology for registration would bias the evaluation of MR biomarkers against the histological response. Additionally, evaluating the registration from landmarks directly between in vivo MR and histology might not accurately evaluate the accuracy of registration because the selected features may not correlate. To address this problem, we use different landmarks for each stage for registration evaluation as the correlation between two stages is better defined. Although our method may include treatment features, we minimize the dependence to one stage and only between images of the same MR modality. Using intermediate imaging makes the presented registration workflow applicable for not only improving registration in prior applications, but also validating of a wider range of applications, including MRgFUS ablations in the liver, breast, or kidneys.

A direct comparison between registration methods is not possible due to large differences in the overall workflows and differences in the included anatomies. Prior registration methods often rely on a 2D correlation between in vivo MR and histology^10,20–26^. However, this study emulated the 2D slice correspondence assumption in our ex vivo registration step and compared the error of the presented method against this assumption to demonstrate the advantage of the 3D block reconstruction method. Prior studies have shown that this 2D slice correlation assumption limits the accuracy of the registration to a minimum of $\sim$ 1mm^12^. We find similar limitations to this assumption in our results. For our pipeline, the 2D slice assumption introduces significant amounts of error into the registration pipeline, as shown in Fig. 4. Although the X and Y dimension error could be improved, the amount of unrecoverable error introduced in the Z dimension using the 2D assumption is still significantly (p $=$ 0.015) more than the total error using our novel block reconstruction method. The high registration accuracy, novel block reconstruction method, and independence of correlating MR and histology features clearly show the advantages of the presented multi-stage registration workflow.

The presented registration workflow and metric evaluation do not come without limitations. The time required for tissue processing and data reconstruction is not trivial. During microtome sectioning (D3), sequentially sectioning through each tissue block while acquiring digital blockface images every 50 $\upmu$m is currently time consuming. In this work, each tissue block must be fully sectioned in order to obtain a 3D model for the ex vivo registration step (R2). However, there are some methods to generate 3D models of the tissue blocks that are more automated and less time intensive, such as micro-CT. Micro-CT can be used to extract the 3D surface of each tissue block without sectioning, leaving the amount of microtome sectioning and blockface imaging up to the discretion of the user. The postprocessing relies on semi-automatic segmentation of blockface imaging and histology sections, which is time consuming at the high resolutions needed to generate high-fidelity models. However, the neural networks used to provide initial segmentations in this study will become more accurate with additional samples, leading to more automatic processing. Additionally, with registration implementation in PyTorch for GPU acceleration, the registration and 3D reconstructions steps only take a few minutes each (R1, R2, R3). This time is expected to be substantially reduced with improved preprocessing and is the subject of ongoing work^33^. Although the presented registration workflow requires more time compared to other workflows, the improved registration accuracy results in a reduction of the necessary number of samples to effectively evaluate MR biomarkers^10,12,26^. Although the small sample size limits the statistical significance of the NPV evaluation, this work demonstrates how the presented registration workflow can be used to rigorously evaluate any kind of MR biomarkers. Future studies utilizing the presented registration method can be added to these results to improve the statistical power of the biomarker evaluation. Additionally, as more data becomes available, current and novel biomarkers can be prospectively studied to fully understand their accuracy for predicting histology necrosis.

Application of the presented workflow to preclinical MRgFUS treatment models will inform the development and validation of data-driven MR biomarkers for future clinical use. The purpose of this study was to develop a novel registration method that could be applied to all MRgFUS treatment regions, facilitating registration of MR and histology outcomes for various MRgFUS applications that was not possible before. However, this work was developed and evaluated in a preclinical model and would need to be altered in future work to be incorporated into existing clinical standard of care workflows, in particular pathology gross room procedures. However, successful integration with current clinical workflows would allow corroboration of the presented preclinical findings, providing a method to validate MR imaging biomarkers for developing and existing MRgFUS treatment procedures.

Despite these limitations, the presented registration workflow facilitated extensive comparisons between the commonly utilized MRgFUS NPV biomarker and gold-standard histology for viability. Contextualize the meaning of the precision and recall scores for MRgFUS ablation treatments of oncological targets is important. If an MR biomarker has low precision with high recall, such as the acute NPV biomarker, a large number target pixels may be labeled as treated, although they are still viable. Relying solely on these MR biomarkers may result in untreated cancer remaining after MRgFUS ablation. Although the acute NPV biomarker is not optimal for immediate MRgFUS treatment assessment, several promising acute MR biomarkers will need to be evaluated against histology, including T2w imaging, MR temperature imaging, and diffusion imaging^13–17^. The presented registration methods can facilitate evaluating and validating these proposed biomarkers in future studies. It should also be noted, that while the presented registration technique has been evaluated in a MRgFUS ablation treatment model, this technique could be potentially applied in any tissue to evaluate a variety of therapeutic options, extending its utility beyond MRgFUS.

We presented a novel, multi-step MR to histology registration workflow that corrects all deformations without assuming any feature correlation between in vivo MR and histology, but rather between intermediate imaging steps. The accuracy of the presented registration workflow has a lower error than previously reported state-of-the-art in vivo MR to histology registration methods. The novel contribution of our presented workflow is the block reconstruction step depicted in Fig. 2. The presented registration workflow facilitated novel spatially accurate evaluation of MRgFUS NPV biomarkers against the gold-standard label of tissue viability and can be used to evaluate and develop novel, acute in vivo MR metrics that can more accurately predict the tissue viability measured with histology. Validating in vivo MR biomarkers will increase their clinical viability for MRgFUS treatment assessment and facilitate clinical translation of minimally invasive treatments for oncological applications.

## Methods

The presented MR to histopathology registration pipeline was evaluated in a large animal tumor model. Supplemental Video 1 provides an animation of the entire tissue processing and reconstruction workflow. To evaluate both the acute NPV and the post-NPV as MR imaging biomarkers, the acute NPV was registered to the post-NPV using methods described in Zimmerman et al.^34^ The tissue from the subject was excised immediately after follow-up imaging according to the procedures outline in “D1: Tissue excision”, “D2: Gross slicing” and “D3: Microtome sectioning”. The histology was reconstructed and registered to the follow-up in vivo MR images according to the procedures outlined in “R1: Histology to block registration”, “R2: Tissue blocks to ex vivo registration” and “R3: Ex vivo to in vivo registration”.

### Subject model

A VX2 cell suspension ($1\times 10^{6}$ cells in 50% media/Matrigel) was bilaterally injected intra-muscularly into the quadriceps of four New Zealand white rabbits and grown for 1–2 weeks. Anesthesia was induced with a ketamine (Zetamine 100 mg/ml from VetOne)/xylazine (AnaSed LA 100 mg/ml from VetOne) injection (IM, 25/5 mg/kg), and the animal was then intubated, allowing anesthesia to be maintained with inhaled isoflurane (0.5–4.0%) for the duration of the MRgFUS treatment. Animal vitals, including temperature and respiration, were monitored throughout treatment. Hair on the treated quadriceps was removed via clippers and a depilatory cream (Nair) to enable acoustic coupling. Using a pre-clinical MRgFUS system (Image Guided Therapy, Inc.), ablation was performed on one tumor and the surrounding quadriceps muscle tissue with a 256-element phased-array transducer (Imasonic, Voray-sur-l’Ognon, France; 10-cm focal length, 14.4 $\times$ 9.8 cm aperture, f $=$ 940 kHz) inside a 3T MRI scanner (PrismaFIT Siemens, Erlangen, Germany). Ablation details for the four subjects are outlined in Table 2. A single loop MR receiver coil was incorporated into the MRgFUS system table to improve the image signal to noise ratio (SNR) around the targeted quadriceps. The ablation procedure was monitored in real time with MR thermometry imaging (MRTI) using 3D-segmented echo planar imaging sequences. Following ablation therapy, the animal was recovered and monitored for 3 days. After 3 days, the animal was re-anesthetized and follow-up (post) imaging was performed. For more details on the in vivo MR imaging, we refer the reader to Zimmerman et al.^34^ The animal was euthanized immediately following imaging. The study was carried out in compliance with the Animal Research: Reporting of In Vivo Experiments (ARRIVE) guidelines. The University of Utah Institutional Animal Care and Use Committee (IACUC) approved all procedures (#17-08012, September 7, 2017). All methods and procedures were performed in accordance with the IACUC guidelines and regulations.

**Table 2:** Ablation details for each subject including time between treatment MR (where the acute NPV is measured) and follow-up imaging (where post-NPV is measured), ablation points, acoustic power output, and total energy achieved for each subject.

#.	Follow-up duration	Number of sonications	Acoustic power Mean ± 1 std. (W)	Total energy (kJ)
1	5 days	11	57 ± 17	23.14
2	3 days	12	69 ± 18	26.00
3	5 days	14	44 ± 9	18.59
4	5 days	10	56 ± 9	18.55

### D1: Tissue excision

The treated quadriceps was surgically excised immediately following euthanization. Pathology inks were applied to the tissue during excision to maintain the MR orientation, and the excised tissue was submerged in 10% formalin solution for 14 days. The fixed ex vivo tissue was mounted in a custom tissue-processing box and encapsulated in 3.5% agar solution for ex vivo imaging and subsequent gross slicing. The custom box and pathology inks facilitated orienting the tissue as close to the in vivo MR orientation as possible. The tissue-processing box incorporated a single-loop MR coil around the box to improve ex vivo MR image SNR. T1w and T2w MR images of the agar-embedded tissue were acquired using a 3T MRI scanner (PrismaFIT Siemens, Erlangen, Germany). The field of view and voxel size for in vivo and ex vivo T1w MR images used in the registration pipeline was 256 $\times$ 56 $\times$ 192 mm with 0.5 $\times$ 1.0 $\times$ 0.5 mm spacing, and for T2w MR the field of view and spacing was 256 $\times$ 52 $\times$ 192 with 1.0 mm isotropic spacing.

### D2: Gross slicing

Following ex vivo imaging, the agar embedded tissue block was grossly sliced along the head-foot axis of the *ex vivo* MR imaging in $\sim$3 mm increments using a deli slicer (Backyard Pro SL110E). The surrounding agar was removed from each sliced tissue block without disturbing the tissue blocks. The exposed tissue faces from gross slicing were re-inked with pathology inks to indicate the head and foot surfaces and maintain the orientation of each block relative to the original ex vivo sample. Each tissue block was placed into an individual whole-mount tissue cassette for further formalin fixation (5 days). After the additional fixation, each block was embedded in paraffin wax with consistent orientation.

### D3: Microtome sectioning

Each paraffin wax tissue block was sectioned at 10 $\upmu$m increments with a microtome (Leica RM2255, Leica Microsystems, Wetzlar, Germany). Digital images of the blockface were acquired every 50 $\upmu$m starting from the very beginning of the block using a digital single-lens reflex (DSLR) camera (Nikon D7100; Macro 1:1 105 mm Lens; 2.0$\times$ teleconverter). The image size and approximate resolution for a blockface image are $6000 \times 4000$ with $\sim 0.018$ mm isotropic spacing. Paraffin wax is slightly transparent, so tissue from behind the exposed face would show through on the images. To address this, two images were acquired at every 50 $\upmu$m with two different lighting conditions. The first image had the light approximately aligned with the camera whereas the second image had the light perpendicular to the camera. Taking the difference between two lighting conditions shows only the tissue that is exposed on the surface. These blockface images were acquired automatically with an Arduino board controlled with a Python GUI. The Python GUI automatically triggered the camera and lights, transferred files to the computer, recorded image information (name, section depth, camera settings, etc), and backed up each image to cloud storage to ensure data retention. Three 5 $\upmu$m thick tissue sections were retained on glass slides for future histopathology analysis every 250 $\upmu$m. Sequential sampling was repeated until there was no tissue remaining in the paraffin wax. After sectioning, the first section of each group of three was stained with hematoxylin and eosin (H&E). The remaining sections were reserved for additional future stains. All H&E stained sections were imaged with a brightfield microscope (Axio Scan, Zeiss, Oberkochen, Germany) at 2.5 magnification. The native resolution of the microscopic images is $\sim 0.0076$ mm, but the images were down-sampled to the resolution of the blockface images for use in registration. Labels of necrotic tissue on the H&E stained sections were semi-automatically generated using Gaussian mixture modeling and expert manual segmentation.

### R1: Histology to block registration

Each histopathology section was registered to the nearest incremental 50 $\upmu$m blockface image. The digital histology images were down-sampled to the resolution of the blockface image ($\sim$ 0.02 $\times$ 0.02 $\times$ 0.05 mm). An affine transform between the histology and blockface was solved via automatic intensity-based affine registration of the blockface segmentation and the histology segmentation. Following affine registration, a multi-scale, intensity-based registration was used to deformably register the histopathology segmentation images to the corresponding blockface image segmentations. Each image was registered by minimizing the following energy *E*:

1 $$\begin{aligned} E = \int _{\Omega } \left\Vert I_1(\varphi ^{-1}(\vec {x}, t)) - I_0(\vec {x})\right\Vert ^2, \end{aligned}$$

where $I_1$ is the histopathology segmentation, $I_0$ is the blockface image segmentation, $\varphi ^{-1}(\cdot )$ is a diffeomorphism, and $\Omega$ is the image domain. Equation (1) was optimized using a gradient flow algorithm with a Cauchy–Navier operator^35^. This registration step provided a diffeomorphism between a histology section and its corresponding blockface image that corrected deformations introduced during microtome sectioning.

### R2: Tissue blocks to ex vivo registration

Surface registration techniques were used to drive the restoration of the blocks to their original morphology and account for deformations from gross slicing. A 3D surface, represented by a triangular mesh object, was constructed for each tissue block and the ex vivo tissue from their segmentations using a marching cubes algorithm. The exterior tissue surface was segmented from ex vivo MR to generate a 3D surface of the ex vivo tissue that was used as the target for block reconstruction. For each tissue block, the 2D blockface images were stacked to create a 3D blockface volume. Automatic intensity-based 2D affine registration was used to register sequential blockface images to account for small shifts in camera position during imaging. Finally, blockface volumes were semi-automatically segmented using a custom-trained 3D V-Net neural network and manual segmentation to generate the 3D surfaces of each tissue block^36^. Semi-automatic segmentation was achieved by using the network to generate an initial 3D segmentation that was subsequently corrected by manual segmentation.

**Figure 5:**
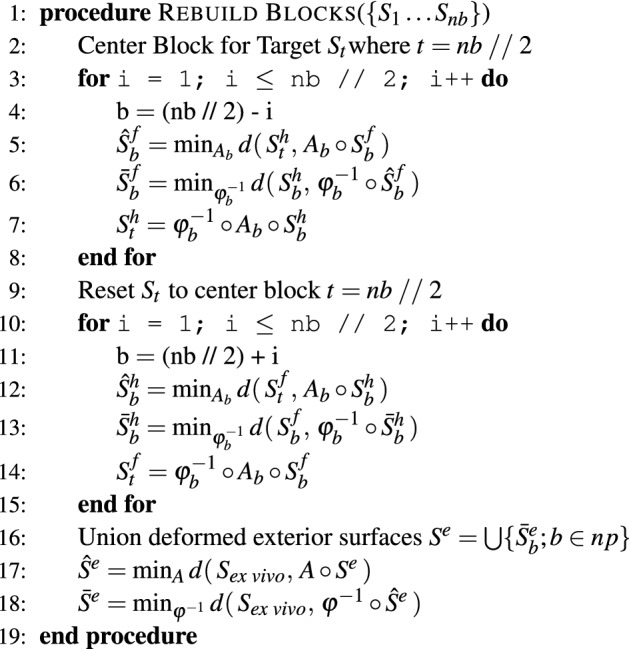
Algorithm overview for reconstruction of the tissue blocks to the ex vivo tissue surface.

Each tissue block was represented by a surface, $S_b$ where *b* indicates the block number ranging from one to the number of total blocks from the gross slicing, *nb*. For a single tissue block, the mesh was semi-automatically separated using surface normals and manual segmentation into a head surface $S_b^h$, foot surface $S_b^f$, and exterior surface $S_b^e$, such that $S_b = S_b^h\cup S_b^f\cup S_b^e$. Examples of the corresponding faces used for registration are shown in Fig. 2b. The corresponding surfaces were registered together using surface-based registration, which is outlined in “Surface-based registration”. The algorithm in Fig. 5 provides an overview of the block reconstruction to ex vivo process. $d(\cdot , \cdot )$ refers to the surface distance metric defined in “Surface-based registration”. In general, a center block was chosen as the starting point for the reconstruction, and deformations were solved for block by block, propagating outwards from the center block first in the head direction, then in the foot direction. Once the deformation was determined for one block, the deformed block then became the target of registration for the next sequential block. Each block had an associated affine transformation and diffeomorphism, $A_b$ and $\varphi ^{-1}_b$, respectively. After all blocks were registered and stacked back together, the deformed exterior surfaces from each block were joined together and registered with the ex vivo tissue surface.

### R3: Ex vivo to in vivo registration

Expert segmentations of corresponding features, such as blood vessels, tumor, or treatment features, identified from the T2w images from the ex vivo and *in vivo* MR data were used to generate 3D surfaces in both spaces. The tissue was excised immediately following the collection of the in vivo images, so the assumption is that features present in the ex vivo images directly correlate to those in the in vivo images. As a result, any difference in the shape or presentation of these features is due to deformations introduced during the tissue excision and tissue fixation steps. Therefore, to correct for the deformations between these imaging stages, an affine transform and diffeomorphism between ex vivo and in vivo MR was solved using 3D surface-based registration defined in “Surface-based registration”.. The full transformation from the in vivo to ex vivo environment was comprised of an affine matrix and a diffeomorphism. All deformations from the three restoring registration pipeline steps were composed to yield a final diffeomorphism that was a spatial mapping between in vivo MR and any histopathology section.

### Surface-based registration

Surface-based registration was used throughout the reconstruction pipeline to solve for affine transforms and diffeomorphisms between two 3D surfaces. The surfaces did not have corresponding landmarks that could be identified from the images or surfaces. We implemented a previously developed unlabeled point-set matching algorithm to solve for a diffeomorphism between the two surfaces^37^. All surface objects were defined as triangular mesh objects. For a single triangular mesh object *S*, the kernel norm (K-norm) is defined as

2 $$\begin{aligned} \left\Vert \sum _{p=1}^{n \in S} \eta (p) \delta _{c(p)}\right\Vert _K^2 = \sum _{p=1}^{n} \sum _{p'=1}^{n} \; \langle \eta (p), \eta (p') \rangle \; K(c(p),c(p')) \end{aligned}$$

where *p* is a triangular element of *S*, $n \in S$ is the number of faces in surface *S*, $\eta (p)$ the normal vector to *p*, *c*(*p*) is the center point of triangle *p*, $K(\cdot , \cdot ) = k(\cdot , \cdot )I$ where *I* is a $3\times 3$ identity matrix and $k(\cdot , \cdot )$ is a scalar valued Cauchy kernel. The dissimilarity between two surfaces $S_1$, $S_2$ is driven by the difference between the sum of the vector valued Dirac masses centered at the triangle center with

3 $$\begin{aligned} \begin{aligned} \left\Vert \sum _{p=1}^{n \in S_1} \eta (p) \delta _{c(p)} - \sum _{q=1}^{m \in S_2} \eta (q) \delta _{c(q)}\right\Vert _K^2&= \sum _{p=1}^{n} \sum _{p'=1}^{n} \; \langle \eta (p), \eta (p') \rangle \; K(c(p),c(p')) \\&\quad - 2 \sum _{p=1}^{n} \sum _{q=1}^{m} \; \langle \eta (p), \eta (q) \rangle \; K(c(p),c(q)) \\&\quad +\sum _{q=1}^{m} \sum _{q'=1}^{m} \; \langle \eta (q), \eta (q') \rangle \; K(c(q),c(q')). \end{aligned} \end{aligned}$$

For a more detailed explanation on how affine transformations and diffeomorphisms act on these surfaces objects, we refer the reader to Glaunes et al.^37^.

### NPV biomarker analysis

After performing registration, the acute NPV biomarker, post-NPV biomarker, and the histology necrosis label were co-registered in the follow-up MR imaging space (where post-NPV was measured). The spatial accuracy of the acute NPV and the post-NPV biomarkers were evaluated against the histology necrosis label using precision, recall, DICE coefficient, and Hausdorff distance. The acute NPV vs histology and post-NPV vs histology spatial metrics were compared using two-sample-independent t-tests.

### Registration accuracy

Landmarks were used to validate the accuracy of the registration via the target registration error (TRE). Selecting landmarks directly between MR and histology is difficult for two reasons: first, selecting landmarks between 2D and 3D spaces is challenging, and the relationship between MR treatment features and histology treatment features is being evaluated and cannot be used to evaluate the registration. Consequently, we selected three sets of N $=$ 20 landmarks (five per subject) for each of the four subjects to evaluate each step of the registration pipeline individually. The TRE was calculated via the Euclidean distance between the deformed landmarks and the target landmarks for each stage of registration. The upper bound of the total registration pipeline was estimated by summing the mean error from each individual stage.

The novel contribution of our registration pipeline is the 3D block reconstruction to insert each histology slice into the 3D ex vivo MR space. Prior registration methods assumed a 2D relationship between 2D histology and a 2D MR slice. We emulate this assumption during our 3D block reconstruction by restricting the Z dimension (perpendicular to the histology sections) block motion to only translation, effectively assuming that the 2D histology slices align with a 2D ex vivo MR slice. Using the landmarks, we calculate the TRE under this 2D projection assumption to demonstrate the improved accuracy of the presented 3D block reconstruction. The TREs of the two different methods were compared using two-sample-related t-tests on the Euclidean error between the deformed landmarks and target landmarks.

## Supplementary Information

Supplementary Legends.

Supplementary Video S1.
