# Supplementary material for: Histology to 3D in vivo MR registration for volumetric evaluation of MRgFUS treatment assessment biomarkers
Source: Sci Rep. 2021 Sep 23;11:18923. doi: 10.1038/s41598-021-97309-0 (PMC8460731; doi:10.1038/s41598-021-97309-0)
Supplement: Supplementary file 1 — Supplementary Legends. [file 41598_2021_97309_MOESM1_ESM.pdf]

Histology to 3D *In Vivo* MR Registration for Volumetric Evaluation of MRgFUS Treatment Assessment Biomarkers

Authors: Blake E. Zimmerman, Sara L. Johnson, Henrik A. Odéen, Jill E. Shea, Rachel E. Factor, Sarang C. Joshi, and Allison H. Payne

Please find Supplementary video 1 in the attached “Supplementary Video 1.zip” file. This video provides an animation of the entire tissue processing and reconstruction workflow.
